# Efficacy and safety of modified-dose obinutuzumab in the treatment of refractory membranous nephropathy

**DOI:** 10.3389/fimmu.2026.1787013

**Published:** 2026-04-22

**Authors:** Jiannan Zhou, Sanxi Ai, Xin Zhang, Xiao Wang, Gang Chen, Yiping Wang

**Affiliations:** 1 Department of Nephrology, Beijing Aerospace General Hospital, Beijing, China; 2 Department of Nephrology, Peking Union Medical College Hospital, Chinese Academy of Medical Sciences & Peking Union Medical College, Beijing, China; 3 Renal Division, Department of Medicine, Peking University First Hospital, Beijing, China

**Keywords:** B-cell depletion, obinutuzumab, PLA2R antibody, refractory membranous nephropathy, rituximab-resistance

## Abstract

**Introduction:**

Primary membranous nephropathy (MN) is an autoimmune kidney disease, and the main autoantibody is directed against the M-type phospholipase A2 receptor (PLA2R). Approximately 20-40% of patients with MN do not respond to standard immunosuppressive therapy, termed refractory MN. Previous studies suggest obinutuzumab’s efficacy in refractory MN, but the optimal dosing regimen remains undefined.

**Methods:**

This single-center retrospective study investigated the efficacy and safety of a B-cell reconstitution-guided regimen of obinutuzumab in patients with refractory MN. All patients were followed at 3-month intervals for up to 18 months. The primary outcome was a composite of complete or partial remission. The secondary outcomes included complete remission, B-cell depletion, immunological remission, and safety profiles.

**Results:**

A total of 33 patients with refractory MN were included (81.8% were rituximab-refractory), with a mean age of 46.1 ± 12.1 years. At baseline, these patients had a mean 24-hour urinary protein of 6.7 ± 4.9 g, a mean serum albumin of 30.0 ± 8.3 g/L, and positive anti-PLA2R in 19 (57.6%) cases. The median total obinutuzumab dose was 2 g (IQR: 2-3). Following treatment, the cumulative remission rate during the 18-month follow-up period reached 84.8%. B cells were depleted in all patients at month 3, and the median duration of sustained B-cell depletion was 12 months (95% CI: 9.5-14.5). Among the 19 anti-PLA2R-positive patients, 84.2% achieved immunological remission at month 18. No patients experienced severe adverse events.

**Conclusion:**

This study demonstrates that B-cell reconstitution-guided modified-dose obinutuzumab is associated with favorable efficacy and a safety profile in refractory MN. The individualized strategy minimized drug exposure while maintaining efficacy, but the conclusions warrant further validation in controlled studies.

## Introduction

Primary membranous nephropathy (MN) remains the leading cause of nephrotic syndrome in nondiabetic adults, characterized by the formation of subepithelial immune complexes (1, 2). It is an autoimmune kidney disease, and the primary autoantibody involved is directed against the M-type phospholipase A2 receptor (PLA2R) (3). The therapeutic landscape of MN has evolved significantly with the adoption of B-cell depletion therapies, represented by rituximab. Rituximab is a chimeric type I anti-CD20 monoclonal antibody, and has been recommended as a first-line treatment for primary MN due to its proven efficacy and safety (4–6). Despite these advances, approximately 20% to 40% of patients with MN fail to achieve remission with rituximab or traditional immunosuppressive regimen, a condition termed refractory MN (4–7). For these patients, persistent nephrotic-range proteinuria confers a substantial risk of progression to end-stage renal disease and complications related to nephrotic syndrome.

Obinutuzumab is a humanized type II anti-CD20 monoclonal antibody with a glycol-modified Fc. It exhibits superior B-cell depletion efficacy than rituximab due to distinct mechanistic advantages, including reduced internalization and degradation, enhanced Fc gamma receptor-mediated effector functions, superior direct cell death induction, and reduced immunogenicity (8, 9). Evidence from case series and small cohort studies suggests the efficacy of obinutuzumab for refractory MN, especially rituximab-refractory MN (10–14). However, the optimal dosing regimen remains to be established. The most common dosing regimen for obinutuzumab includes an initial course of two 1-gram doses administrated 2 weeks apart, with potential additional doses based on treatment responses (10–12). Additionally, some protocols have utilized an initial course of three 1-gram doses, with intervals of either 1 or 2 weeks between administrations (13, 14). These dosing schedules are adapted from experiences in hematologic malignancies, which often employ fixed doses of 1000 mg on days 1, 8, and 15.

Given the central role of B cells in the production of pathogenic autoantibodies in MN, a treatment strategy aimed at B cell depletion represents a rational therapeutic approach. We hypothesize that in an autoimmune setting, sustaining B-cell depletion is more critical than achieving the high peak drug concentrations required for tumor lysis. Therefore, an individualized dosing strategy guided by B-cell kinetics may theoretically maintain efficacy while minimizing drug exposure. This retrospective study evaluates the efficacy and safety of a B-cell reconstitution-guided, modified-dose obinutuzumab regimen in a cohort of patients with refractory MN.

## Methods

### Participants

This is a single-center, retrospective study conducted in Beijing Aerospace General Hospital, China, from October 2022 to August 2023. Inclusion criteria were as follows: (1) MN diagnosed by renal biopsy or serological assay (PLA2R antibody titer ≥20 RU/mL); (2) primary MN diagnosed after excluding concurrent autoimmune diseases, malignancies, and infections (including hepatitis B and C, human immunodeficiency virus, and syphilis); (3) refractory MN, defined as failure to achieve clinical remission after at least 6 months of standard immunosuppressive therapy including corticosteroids combined with cyclophosphamide, calcineurin inhibitors, or rituximab. Patients were excluded if they had a diagnosis of diabetes mellitus or if they were unwilling to participate in the study. Rituximab-refractory MN was defined as absence of clinical and/or immunological remission during a follow-up period exceeding 6 months after at least 2 doses of 1g rituximab administration (10, 11, 15). This study was approved by the Ethics Committee of Beijing Aerospace General Hospital. Written informed consent was obtained from all participants.

### Treatment and follow-up

Patients received obinutuzumab (Roche, South San Francisco, CA) as an intravenous infusion, with an initial dose of 1 g. Repeat dosing followed a predefined protocol: retreatment with an additional 1g infusion was triggered when CD19^+^ B-cell count reconstituted to ≥ 5/μL (B-cell reconstitution), unless complete remission had already been achieved. This criterion was applied regardless of anti-PLA2R titers. Each infusion was preceded by premedication (5 mg dexamethasone and 25 mg promethazine) and were performed under continuous cardiac monitoring for adverse event surveillance. Following obinutuzumab initiation, concomitant corticosteroids and calcineurin inhibitors and cyclophosphamide were tapered and discontinued at the discretion of the attending physicians. All patients received optimal supportive therapy, including the maximum tolerated dose of an angiotensin-converting enzyme inhibitor or angiotensin receptor blocker, as well as lipid-lowering drugs, prophylactic antithrombotic treatments, diuretics as needed. Prophylactic entecavir was initiated in all patients with evidence of prior HBV exposure (positive HBsAg or positive HBcAb) before obinutuzumab administration. All patients received prophylaxis against Pneumocystis jirovecii pneumonia with trimethoprim-sulfamethoxazole during immunosuppressive therapy, unless contraindicated.

All patients received regular follow-up with comprehensive assessments every three months for up to 18 months. Assessments included serum creatinine, eGFR, serum albumin, CD19+ B cell counts, PLA2R antibody titers, and 24-hour urinary protein excretion. The eGFR was calculated via the Chronic Kidney Disease Epidemiology Collaboration creatinine equation (16). CD19+ B cell counts were quantified by flow cytometry (Beckman Coulter DxFLEX). Anti-PLA2R antibody titers were measured by time-resolved fluorescence immunoassay using Biostar TRF-1000 analyzer. Adverse events such as cardiovascular events, thrombotic events, infections, allergic reactions were documented throughout the treatment and follow-up period.

### Outcomes and definitions

All patients were followed up for 18 months. The primary outcome was clinical remission which was defined as a composite of complete or partial remission over the 18-month follow-up period. According to the Kidney Disease Improving Global Outcomes guidelines (17), complete remission was defined as 24-hour urine protein < 0.5 g, serum albumin ≥ 35 g/L, and < 25% eGFR decline from baseline. Partial remission was defined as 24-hour urine protein < 3.5 g with a > 50% decrease from baseline, serum albumin ≥ 30 g/L, and <25% eGFR decline. Relapse was defined as the development of urinary protein >3.5 g per 24 hours after achieving complete or partial remission.

The secondary outcomes included complete remission, B-cell depletion, immunological remission, and safety profiles. Immunological remission was defined as anti-PLA2R antibody levels < 2 RU/mL in anti-PLA2R-positive patients. B-cell depletion was defined as peripheral CD19^+^ cell count < 5/μL.

### Statistical analysis

Categorical variables were described as frequencies and percentages, and were compared using chi-square test or Fisher exact test. Continuous variables were presented as mean ± standard deviation for normally distributed data or median (interquartile range) for non-normally distributed data. Comparison between remission and non-remission groups were performed using Student’s t-test or the Wilcoxon signed-rank test as appropriate. Kaplan-Meier analysis was employed to estimate the cumulative probability of clinical remission. All tests were two-tailed, with the threshold for statistical significance set at P < 0.05 (α=0.05). Statistical analyses were conducted using SPSS software (version 25.0, IBM) and GraphPad Prism (version 11.0).

## Results

### Baseline characteristics

**Table 1** shows the baseline disease characteristics of the patients. The study included 33 patients with refractory primary MN, with a mean age of 45.9 ± 12.3 years (27 males, 6 females). Among the 33 patients, 14 patients were diagnosed by renal biopsy. However, as many of these biopsies were performed at external centers, information regarding PLA2R antigen positivity and the specific extent of tubulointerstitial injury was unavailable.

**Table 1:** Baseline characteristics and obinutuzumab doses.

Parameters	All patients (n=33)	Remission (n=28)	Non-remission (n=5)	P value
Male, n (%)	27 (81.8%)	23 (82.1%)	4 (80.0%)	
Age (years)	45.9 ± 12.3	48.0 ± 11.8	34.4 ± 8.6	0.01
Disease duration (months)	66.9 ± 49.5	60.4 ± 45.1	103.2 ± 62.7	0.07
Previous immunosuppressive treatment	33 (100%)	28 (100%)	5 (100%)	
Glucocorticoid, n (%)	25 (75.8%)	21 (75%)	4 (80%)	0.81
Calcineurin inhibitors, n (%)	33 (100%)	28 (100%)	5 (100%)	> 0.99
Rituximab, n (%)	27 (81.8%)	21 (75%)	5 (100%)	0.56
Cyclophosphamide, n (%)	16 (48.5%)	14 (50%)	2 (40%)	0.68
CKD 3-4, n (%)	15 (45.5%)	13 (46.4%)	2 (40%)	0.79
24h urine protein excretion (g)	6.7 ± 4.9	7.0 ± 5.1	5.1 ± 3.2	0.54
Serum albumin (g/L)	30.0 ± 8.3	29.5 ± 7.8	32.6 ± 11.2	0.51
eGFR (mL/min/1.73 m²)	66.6 ± 29.7	65.6 ± 30.4	71.7 ± 27.4	0.68
Anti-PLA2R positivity, n (%)	19 (57.6%)	16 (57.1%)	3 (60%)	
Anti-PLA2R titer (RU/ml)	36.0 (21.0, 85.0)	40.3 (30.6, 102.7)	21.0 (17.9, 32.7)	0.21
B-cell count (cells/ul)	28 (6, 112)	29 (8, 122)	10 (2, 64)	0.26
Cumulative dose of obinutuzumab (g)	2.3 ± 1.0	2.2 ± 0.9	3.0 ± 1.0	0.09
1 g, n (%)	6 (18.2%)	6 (21.4%)	0 (0%)	0.25
2 g, n (%)	15 (45.5%)	13 (46.4%)	2 (40%)	0.79
3 g, n (%)	7 (21.2%)	6 (21.4%)	1 (20%)	0.94
4 g, n (%)	5 (15.1%)	3 (10.7%)	2 (40%)	0.09

Twenty-seven (81.8%) patients were rituximab-refractory, who had received a median cumulative dose of 2 g (2, 3 g) of rituximab. All patients had prior exposure to calcineurin inhibitors; 75.8% to glucocorticoids, and 48.5% to cyclophosphamide. The mean disease duration from diagnosis of MN to the initiation of obinutuzumab was 66.9 ± 49.5 months.

At baseline, patients presented with a mean 24-hour urinary protein of 6.7 ± 4.9 g, a mean serum albumin of 30.0 ± 8.3 g/L, and a mean eGFR of 66.6 ± 29.7 mL/min/1.73 m². Fifteen patients had CKD stage 3-4. Immunological profiles revealed a median absolute B-lymphocyte count of 28 cells/µL (6, 112) and anti-PLA2R seropositivity in 19 patients, with a median antibody titer of 36.0 RU/ml (21.0, 85.0). At baseline, 14 patients tested negative for anti-PLA2R antibodies, all of whom were diagnosed with MN by renal biopsy. However, data on tissue PLA2R antigen staining were unavailable for these patients. Among them, 9 had persistently negative anti-PLA2R antibodies, while the remaining 5 had seroconverted to negative prior to obinutuzumab administration.

### Treatment and outcomes

The median interval between the last rituximab dose and obinutuzumab administration was 12 months (range, 6–24 months). The median total obinutuzumab dose was 2 g (IQR: 2-3). Six patients received a single dose of 1 g, fifteen received 2 g, seven received 3 g, and five received 4 g. Nineteen patients stopped immunosuppressants before starting obinutuzumab, 10 patients discontinued immunosuppressants within 6 months after obinutuzumab initiation, and the remaining 4 patients remained on low-dose calcineurin inhibitors for the full 18 months of follow-up. Following obinutuzumab treatment, B-cell depletion was achieved, and a subsequent decrease in 24-hour proteinuria was observed (**Supplementary Figure 1**).

**Figure 1:**
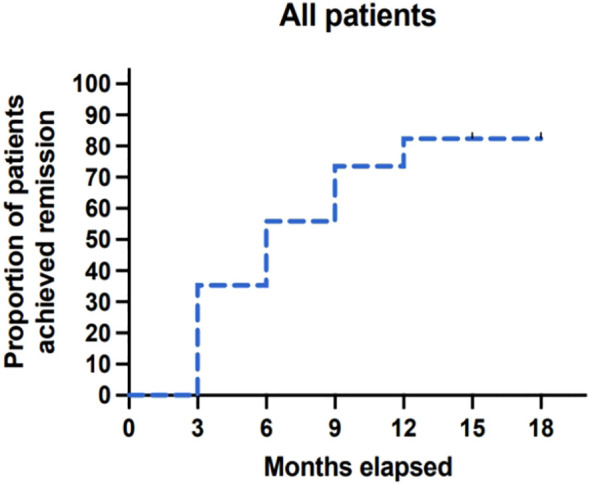
Kaplan-Meier analysis of clinical remission, including complete and partial remission.

By Kaplan-Meier analysis, the cumulative incidence of clinical remission was 84.8% at 18 months of follow-up, and the median time to achieve clinical remission after obinutuzumab treatment was 6 months (95% CI: 6 to 9 months) (**Figure 1**). The cumulative incidence of complete remission was 45.5% by the end of the study. The median time to complete remission was not reached as less than 50% of patients achieved complete remission within the 18-month follow-up period. During follow-up, 7 patients experienced recurrence, 4 of whom achieved remission after re-treatment with obinutuzumab.

**Table 2** shows rates of clinical remission, complete remission, immunological remission, and B-cell depletion at each 3-month visit. Remission rates increased with extended follow-up. At month 3, 36.4% (12/33) of patients had achieved clinical remission (including 6.1% (2/33) with complete remission). At month 18, the rates of clinical remission and complete remission reached 69.7% and 45.5%, respectively. Among the 19 anti-PLA2R positive patients at baseline, 52.6% (10/19) achieved immunological remission at month 3, and 84.2% (16/19) had immunological remission at month 18. The median time to immunological remission was 3 months. B cells were depleted in all patients at month 3, and the median duration of sustained B-cell depletion was 12 months (95% CI: 9.5 to 14.5 months).

**Table 2:** Treatment responses after obinutuzumab treatment.

Follow-up time point	Clinical remission (n=33)	Complete remission (n=33)	Immunological remission (n=19)	B-cell depletion (n=33)
3 months	12 (36.4%)	2 (6.1%)	10 (52.6%)	33 (100.0%)
6 months	13 (39.4%)	4 (12.1%)	13 (68.4%)	27 (81.8%)
9 months	15 (45.5%)	7 (21.2%)	12 (63.2%)	25 (75.8%)
12 months	14 (42.4%)	7 (21.2%)	15 (78.9%)	24 (72.7%)
15 months	20 (60.6%)	7 (21.2%)	15 (78.9%)	24 (72.7%)
18 months	23 (69.7%)	15 (45.5%)	16 (84.2%)	26 (78.8%)

Data are presented as number (percentage). Percentages represent point remission rate at each time point, and are calculated based on the total number of patients analyzed for each endpoint, as indicated in the column headers.

The response rates (partial or complete remission) was comparable across subgroups, including sex, baseline anti-PLA2R status, and CKD stage (stages 1–2 vs. 3-4) (**Supplementary Table 1**). Although patients with CKD stages 1–2 had a numerically higher complete remission rate than those with stages 3-4, the difference was not statistically significant (P = 0.50).

**Figure 2:**
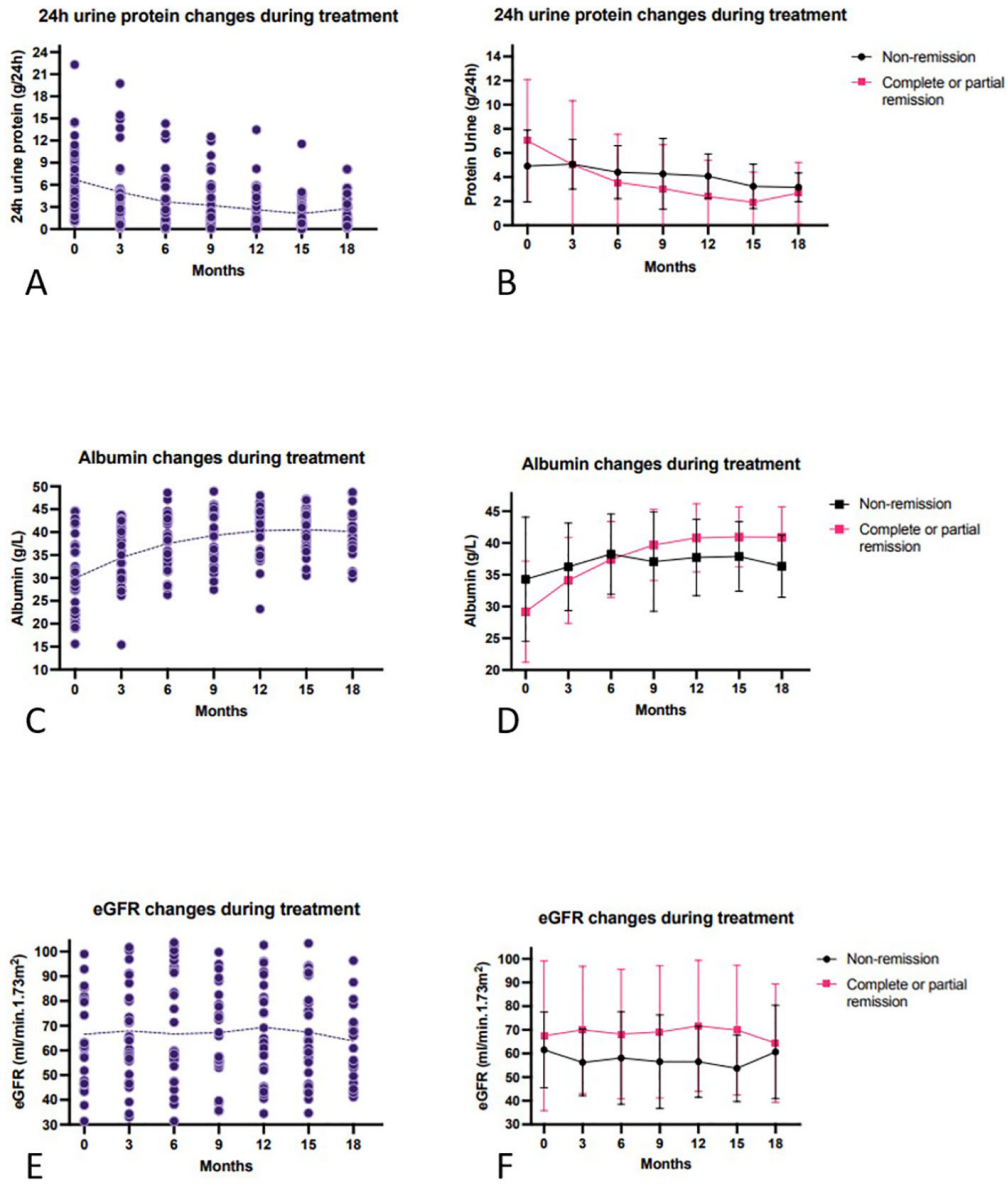
Change of clinical parameters during follow-up. **(A)** 24h urine protein changes in all patients. **(B)** 24h urine protein changes in remission and non-remission group, respectively. **(C)** Serum albumin changes during follow-up. **(D)** Albumin changes in remission and non-remission groups, respectively. **(E)** eGFR changes in all patients. **(F)** eGFR changes in remission and non-remission groups, respectively.

**Figure 3:**
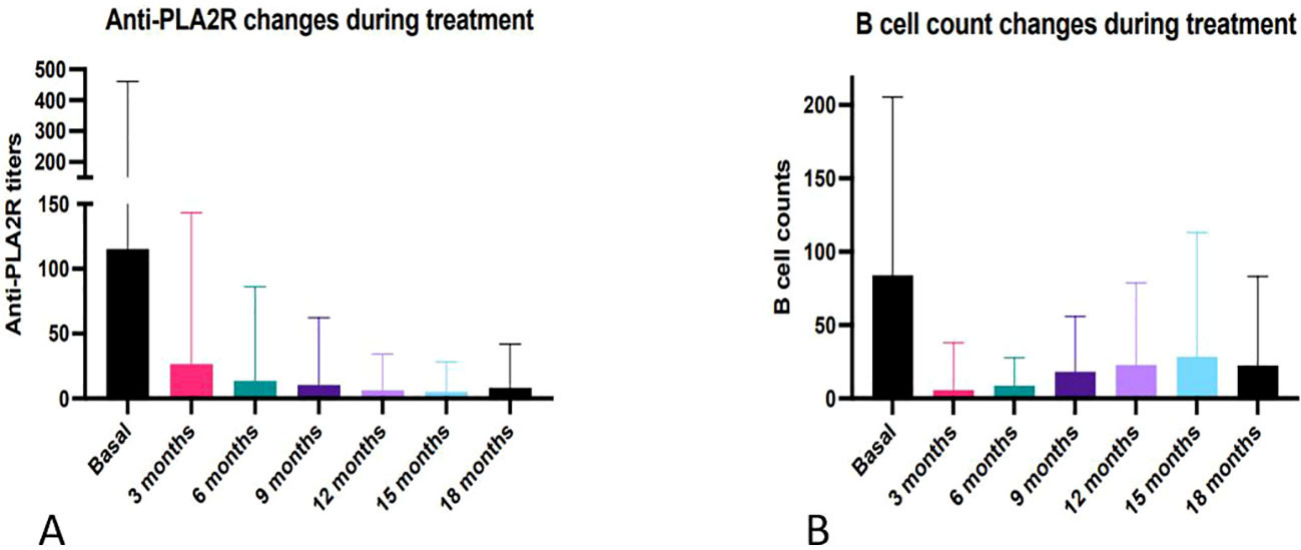
Changes of anti-PLA2R titer and B-cell count during follow-up. **(A)** Change of anti-PLA2R titers in baseline anti-PLA2R-positive patients (n=19). **(B)** B-cell count changes in all patients.

The detailed data of laboratory parameters at each 3-month visit are presented in **Supplementary Table 2**, and the longitudinal trends are graphically represented in **Figures 2**, **3**. At month 3, a significant reduction in 24-hour urinary protein excretion was observed (from 6.7 ± 4.9 to 5.1 ± 4.9 g, p = 0.04). Among the patients who achieved remission, the 24-hour urinary protein level dropped to its lowest point of 1.9 ± 2.5 g at month 15. Serum albumin increased significantly by 3 months post-treatment (from 30.0 ± 8.3 to 34.4 ± 6.7 g/L, p=0.83) and continued to rise until stabilizing after 12 months. eGFR remained stable throughout follow-up, with no significant change from baseline to the last visit (from 66.6 ± 29.7 to 66.6 ± 24.1 mL/min/1.73 m^2^, p > 0.05). Among the 19 baseline anti-PLA2R positive patients, anti-PLA2R titers declined significantly after 18 months of treatment (from 36.0 (21.0, 85.0) to 1.5 (1.0,2.0), p < 0.001).

### Comparison between remission and non-remission groups

The comparison between the remission and non-remission groups was shown in **Table 1**. Compared to the remission group, the non-remission group had longer disease duration (103.2 ± 62.7 vs. 60.4 ± 45.1 months, p = 0.07) and higher cumulative doses of obinutuzumab (3.0 ± 1.0 vs. 2.2 ± 0.9) (p = 0.09), although the differences were not statistically significant. Among the 5 patients who did not achieve remission, four exhibited a 30%–50% reduction in 24-hour urinary protein excretion. Three of the four patients were anti-PLA2R positive at baseline, and they achieved immunological remission during follow-up. During the 18-month period, eGFR remained stable in patients with complete remission, partial remission, or no remission.

### Adverse events

Mild-to-moderate infusion-related reactions occurred in 9 (27.3%) patients. During follow-up, infectious complications developed in 3 (9.1%) patients, including two cases of herpes zoster and one case of COVID-19. No death or progression to end-stage renal disease occurred. One patient developed atrial fibrillation during the infusion, which resolved with treatment and did not recur.

## Discussion

In this retrospective, single-arm cohort study, we demonstrated the efficacy and safety of a B-cell reconstitution-guided, modified-dose obinutuzumab regimen in 33 patients with refractory MN. In this high-risk cohort, predominantly comprising rituximab-refractory MN (81.8%), we observed a significant clinical benefit over an 18-month follow-up, with a cumulative remission rate of 84.8% (28/33). Overall, obinutuzumab was well tolerated, and the infusion-related reactions were not severe.

The recognition of MN as an autoantibody-mediated disease has paved the way for B-cell depletion therapy in MN, such as rituximab (1–3). Rituximab has been recommended to be a first-line therapy for MN due to its comparable efficacy to conventional immunosuppressants (cyclophosphamide-based regimen, calcineurin inhibitors) (17). However, there is a significant unmet need for patients with refractory MN who fail to respond to rituximab or conventional immunosuppressants. Our study confirms the efficacy of obinutuzumab in refractory MN. The 84.8% remission rate observed in our study aligns with the 80-88% remission rate reported in previous retrospective studies for refractory MN (10–13). There is only one published prospective study, as far as we are aware, has reported a remission rate of 80% in 20 patients with rituximab-refractory MN (14). In this study, among the five patients who did not achieve remission, four already exhibited a significant reduction in proteinuria. The lack of clinical remission in these patients, particularly the three who had already achieved immunological remission, could be attributed to both insufficient follow-up time and the known phenomenon that clinical remission often lags behind serological response (17). In addition, 15 of the 28 patients who achieved remission in our study had CKD state 3–4 at baseline, including one with an eGFR as low as 20 mL/min/1.73 m². This finding suggests that obinutuzumab may hold promise for patients with advanced CKD, aiming to slow the decline of renal function. However, due to the relatively short follow-up of this study, the benefit of obinutuzumab on long-term renal outcomes warrants further validation through studies with longer-term observation.

A major methodological strength of this study is the implementation of a regular follow-up schedule. Most previous studies are based on small retrospective cohorts (generally<30 participants), with relatively short and variable duration of follow-up (generally ≤ 12 months). Consequently, the statistical power of these findings are limited (10–13). Despite being a retrospective study, this study’s protocol of regular 3-month follow-up with comprehensive assessments at each visit for all patients provides significant advantages in data quality and analysis. By presenting complete data series similar to a prospective study, we were able to observe the longitudinal trends in clinical response, anti-PLA2R titers, and B-cell counts, offering a dynamic perspective often missing in typical retrospective studies. Moreover, all patients were followed up for a period of 18 months, which is longer than the follow-up duration in most previous studies (10–13).

In this study, over 80% of patients had rituximab refractory MN. Rituximab resistance arises from several factors, notably the development of anti-drug antibodies (due to its murine components) and (18, 19), fundamentally, the internalization of the CD20-antibody complex by B cells. As a type I anti-CD20 antibody, rituximab binding induces CD20 modulation and internalization, leading to rapid lysosomal degradation and reduced bioavailability, which limits its efficiency of B-cell depletion (20, 21). The efficacy of obinutuzumab in rituximab-refractory MN is attributed to distinct molecular mechanisms. Obinutuzumab is a glycoengineered type II anti-CD20 antibody with minimal internalization, ensuring sustained binding to the B-cell surface. Furthermore, obinutuzumab’s afucosylated Fc region increases affinity for FcγRIIIa receptors on effector cells (e.g., NK cells, macrophages), inducing more potent antibody-dependent cellular cytotoxicity. Moreover, obinutuzumab triggers a far more potent, lysosome-mediated direct cell death (8, 9). In our study, the rapid and sustained B-cell depletion observed in all patients confirms the *in vivo* efficacy of obinutuzumab.

A novel finding of this study is the validation of the feasibility of a “B-cell guided” dosing strategy for obinutuzumab treatment in MN. The B-cell driven doses of rituximab has been demonstrated to be as effective as the four 375 mg/m^2^ protocol in MN, while avoiding unnecessary reexposure to rituximab (22). However, to our knowledge, this “B-cell guided” regimen has not been validated for obinutuzumab in MN. In this study, by administering subsequent doses only upon B-cell reconstitution (≥ 5 cells/μL), the mean cumulative dose over an 18-month period was limited to 2.3 g. The dose used in our study was lower than both the protocol using an initial 2-gram dose (with possible supplements) and the protocol employing an initial 3 grams (10–14). Nevertheless, our regimen successfully induced rapid B cell depletion and immunological remission (median time to immunological remission was 3 months) followed by clinical remission. In addition, it successfully maintained deep B-cell depletion for 12 months. The results suggests that the individualized strategy based on B-cell reconstitution can minimize drug exposure while maintaining efficacy. Nevertheless, due to the small sample size, this conclusion requires validation in larger cohorts. Due to the lack of a direct comparator in the study, we were unable to evaluate the relative merits of this B-cell-driven dosing regimen compared to the existing alternatives.

We acknowledge that the anti-PLA2R-guided regimen is the most accepted dosing protocol for MN. The current guideline also recommended this antibody-driven strategy (primarily anti-PLA2R) in the treatment of MN because the titers of pathogenic antibodies are directly correlated to disease activities (1, 17). The underlying rationale for adopting B-cell reconstitution-based regimen for MN is to eliminate B cells that produce pathogenic antibodies. But the association of B-cell reconstitution with antibody titer and clinical severity in MN is less direct than that of the antibody titer itself, and the evidence supporting B-cell-guided protocol is less robust than that for antibody-guided regimen. Therefore, we support the antibody-guided strategy as the first option in the management of antibody-positive (primarily anti-PLA2R positive) patients. However, in cases where antibodies are negative or undetectable, B-cell constitution-guided dosing regimen can be a optimal individualized strategy.

Notably, the non-remission group received a higher cumulative dose than the remission group (3.0 ± 1.0 g vs. 2.2 ± 0.9 g, p=0.09). This finding argues against a simple dose-response relationship, suggesting instead a ceiling effect in non-responders, where higher doses yield no benefit. The lack of clinical remission in these patients may be driven by B-cell depletion-independent mechanisms, such as chronic glomerular damage. The inference is supported by the fact that the non-remission group had a longer disease duration (103.2 ± 62.7 vs. 60.4 ± 45.1 months, p=0.07) compared to the remission group. Although the small sample size of the non-remission group (n=5) precludes definitive conclusions, this observation may reflect the effect of disease chronicity on treatment responses. A prolonged disease duration may lead to irreversible structural changes in glomeruli, reaching a pathological irreversible point. In such long-standing cases, even if obinutuzumab successfully clears the immunological trigger, residual proteinuria may reflect structural glomerular filtration barrier defects rather than active inflammation. This suggests that for refractory patients with long disease duration, clinical goals should be pragmatic, focusing on delaying renal function decline rather than solely pursuing clinical remission.

This study has several limitations. First, its single-center retrospective design may introduce selection bias and limit the generalizability of the findings to broader populations. Given the small sample size, we were unable to perform multivariable adjustment for potential confounders. Therefore, our findings should be interpreted as exploratory, and future studies with larger cohorts are warranted to confirm these results. Second, the an absence of a control group precludes causal attribution of the observed remission rates to obinutuzumab. Therefore, the findings should be interpreted as preliminary and warrant confirmation in future controlled studies. Third, we evaluated a modified dosing regimen obinutuzumab without an internal comparator, preventing direct comparisons with existing regimens. Future head-to-head comparative studies are warranted. Additionally, anti-rituximab antibody levels were not assessed, which limits the mechanistic interpretation of rituximab refractoriness in this cohort. Finally, the 18-month follow-up, while sufficient for observing remission, is insufficient to assess long-term relapse rates and hard endpoints like end-stage renal disease.

## Conclusion

In conclusion, this study suggests that a B-cell reconstitution-guided dosing regimen of obinutuzumab is associated with favorable efficacy and safety in refractory MN, particularly rituximab-resistant MN. However, due to the absence of a control group, causal conclusions cannot be drawn, and the findings should be interpreted with this limitation in mind. Despite this, the study holds strong translational promise for several reasons. First, it provides preliminary evidence supporting the efficacy of obinutuzumab in therapeutically challenging refractory MN. Second, it highlights a B-cell driven dosing regimen of obinutuzumab that maintains efficacy while reducing drug exposure, potentially translating to cost saving and a lower risk of adverse events. Finally, it provides a viable and complementary option in scenarios where anti-PLA2R is negative or undetectable. Controlled studies are warranted to confirm these findings.

## Data Availability

The original contributions presented in the study are included in the article/**Supplementary Material**. Further inquiries can be directed to the corresponding author.

## Supplementary material

Supplementary Figure 1 Associations between B-cell depletion and 24h urine protein excretion following obinutuzumab treatment.
