# Supplementary figures and images for: Efficacy and safety of modified-dose obinutuzumab in the treatment of refractory membranous nephropathy
Source: Front Immunol. 2026 Apr 22;17:1787013. doi: 10.3389/fimmu.2026.1787013 (PMC13144149; doi:10.3389/fimmu.2026.1787013)

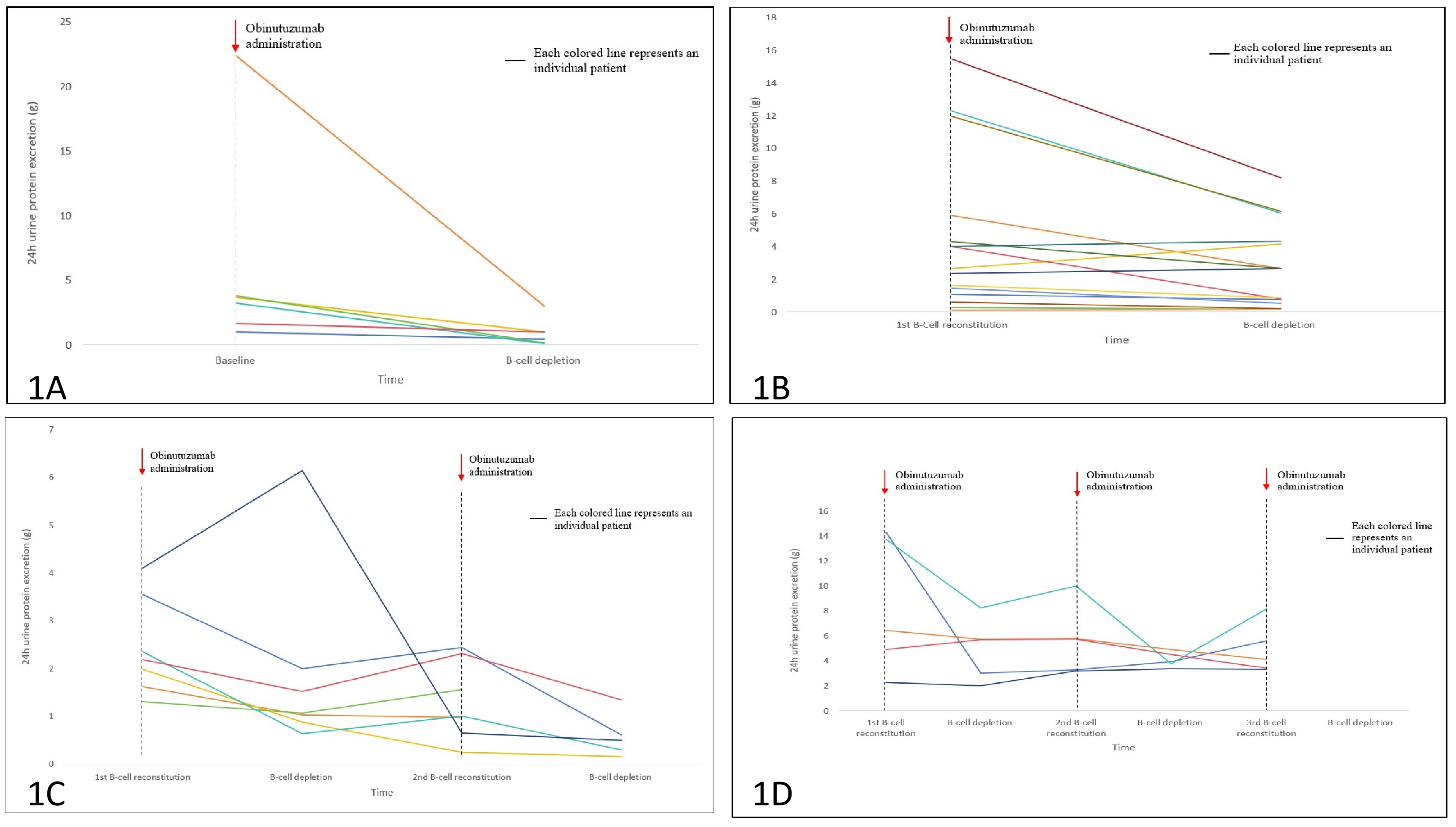

Supplement: Supplementary Figure 1 — Associations between B-cell depletion and 24h urine protein excretion following obinutuzumab treatment. [file Image1.jpeg]
