# Supplementary material for: Efficacy and safety of modified-dose obinutuzumab in the treatment of refractory membranous nephropathy
Source: Front Immunol. 2026 Apr 22;17:1787013. doi: 10.3389/fimmu.2026.1787013 (PMC13144149; doi:10.3389/fimmu.2026.1787013)
Supplement: Supplementary file 2 [file Table1.docx]

Supplemental Table 1：Treatment response in subgroups

| **Treatment response, n (%)** | Anti-PLA2R positive (n=19） | Anti-PLA2R negative (n=14) | P value | CKD stages 1-2 (n=18) | CKD stages 3-4 (n=15) | P value | Male (n=27) | Female (n=6) | P value |
| --- | --- | --- | --- | --- | --- | --- | --- | --- | --- |
| PR or CR | 17 (89.5%) | 11 (78.6%) | 0.39 | 15 (83.3%) | 13 (86.7%) | 0.79 | 23 (85.2%) | 5 (83.3%) | 0.90 |
| CR | 9 (47.3%) | 6 (42.9%) | 0.79 | 11 (61.1%) | 4 (26.7%) | 0.05 | 11 (40.7%) | 4 (66.6%) | 0.37 |
| Immunological remission | 18 (94.7%) | — |  | 9 (100.0%) | 8 (80.0%) | 0.84 | 14 (93.3%) | 3 (75.0%) | 0.38 |

**Supplementary Table 2. Laboratory tests during follow-up**

| Follow-up time points | Parameters | All patients（n=33） | Remission（n=28） | Non-remission (n=5) | P value |
| --- | --- | --- | --- | --- | --- |
| 3 months | 24h urine protein excretion (g) | 5.1±4.9 | 5.2±5.3 | 4.5±2.3 | 0.54 |
|  | Albumin（g/L） | 34.4±6.7 | 34.4±6.6 | 34.9±8.3 | 0.83 |
|  | eGFR（mL/min/1.73 m²） | 67.9±25.7 | 68.3±25.7 | 65.5±28.6 | 0.75 |
|  | Anti-PLA2R titer (RU/ml) | 2.5 (2.0, 16.0) | 2.9 (2.0, 17.3) | 2.0 (2.0, 5.0) | 0.65 |
|  | B-cell count (cells/μl) | 0 (0, 0.2) | 0 (0, 0.3) | 0.5 (0, 139) | 0.45 |
|  |  |  |  |  |  |
| 6 months | 24h urine protein excretion (g) | 3.7±3.8 | 3.5±4.0 | 4.7±2.3 | 0.73 |
|  | Albumin（g/L） | 37.5±6.0 | 37.7±5.7 | 36.8±7.7 | 0.83 |
|  | eGFR（mL/min/1.73 m²） | 66.7±26.3 | 66.3±26.2 | 68.6±30.2 | 0.94 |
|  | Anti-PLA2R titer (RU/ml) | 2.0 (1.8, 3.8) | 2.0 (1.5, 4.2) | 2.0 (1.5, 4.2) | 0.82 |
|  | B-cell count (cells/μl) | 0 (0, 1.7) | 0 (0, 1) | 2 (0.3, 36.0) | 0.54 |
|  |  |  |  |  |  |
| 9 months | 24h urine protein excretion (g) | 3.3±3.5 | 3.1±3.6 | 4.3±2.9 | 0.17 |
|  | Albumin（g/L） | 39.3±5.9 | 39.9±5.3 | 35.8±8.6 | 0.36 |
|  | eGFR（mL/min/1.73 m²） | 67.2±27.0 | 66.9±26.5 | 68.8±33.1 | 0.94 |
|  | Anti-PLA2R titer (RU/ml) | 2.0 (1.8, 5.7) | 2.0 (2.0, 5.8) | 2.0 (1.5, 3.4) | 0.65 |
|  | B-cell count (cells/μl) | 0.2 (0, 32) | 0.1 (0, 4.6) | 0 (0, 36.0) | 0.83 |
|  |  |  |  |  |  |
| 12 months | 24h urine protein excretion (g) | 2.6±3.0 | 2.3±3.1 | 4.0±1.9 | 0.04 |
|  | Albumin（g/L） | 40.4±5.5 | 40.9±5.4 | 37.7±6.0 | 0.23 |
|  | eGFR（mL/min/1.73 m²） | 67.0±25.8 | 66.7±25.7 | 68.8±29.3 | 0.98 |
|  | Anti-PLA2R titer (RU/ml) | 2.0 (1.8, 2.1) | 2.0 (2.0, 2.2) | 2.0 (1.5, 2.0) | 0.43 |
|  | B-cell count (cells/μl) | 0 (0, 20.3) | 0 (0, 1.0) | 0 (0, 1.5) | 0.83 |
|  |  |  |  |  |  |
| 15 months | 24h urine protein excretion (g) | 2.1±2.4 | 1.9±2.5 | 2.9±1.4 | 0.17 |
|  | Albumin（g/L） | 41.1±4.9 | 41.4±4.8 | 39.5±6.1 | 0.58 |
|  | eGFR（mL/min/1.73 m²） | 67.4±26.3 | 67.2±26.1 | 68.8±30.7 | 0.90 |
|  | Anti-PLA2R titer (RU/ml) | 2.0 (1.0, 2.0) | 2.0 (1.0, 2.0) | 2.0 (1.50, 2.0) | >0.99 |
|  | B-cell count (cells/μl) | 0 (0, 24.5) | 0 (0, 1.0) | 0 (0, 0.9) | 0.75 |
|  |  |  |  |  |  |
| 18 months | 24h urine protein excretion (g) | 2.1±2.3 | 2.1±2.4 | 2.1±1.5 | 0.91 |
|  | Albumin（g/L） | 41.1±4.7 | 41.4±4.4 | 39.3±6.6 | 0.64 |
|  | eGFR（mL/min/1.73 m²） | 66.6±24.1 | 65.9±24.2 | 70.9±25.6 | 0.79 |
|  | Anti-PLA2R titer (RU/ml) | 1.5 (1.0, 2.0) | 2.0 (1.0, 2.0) | 1.0 (1.0, 2.0) | 0.49 |
|  | B-cell count (cells/μl) | 0 (0, 15.0) | 0 (0, 18.5) | 0 (0, 8.3) | 0.71 |
